# Supplementary material for: Crawling and Gliding: A Computational Model for Shape-Driven Cell Migration
Source: PLoS Comput Biol. 2015 Oct 21;11(10):e1004280. doi: 10.1371/journal.pcbi.1004280 (PMC4619082; doi:10.1371/journal.pcbi.1004280)
Supplement: S1 Code — (ZIP) [file pcbi.1004280.s012.zip › release/html/index.html]

1-cell CPM


# A single Act cell

This is a simulation of a single Act cell in medium (ECM). The default parameters give rise
to an amoeboid migration pattern. Drag the **Max_act_** slider to a higher
value (e.g., 80) to obtain keratocyte-like migration.

T=0

A_cell_=0

λ_Area_=0

P=0

λ_Perimeter_=0

Max_act_=0

λ_act_=0

J_cell,cell_=0

J_cell,ECM_=0
